# Dense cellular segmentation for EM using 2D–3D neural network ensembles

**DOI:** 10.1038/s41598-021-81590-0

**Published:** 2021-01-28

**Authors:** Matthew D. Guay, Zeyad A. S. Emam, Adam B. Anderson, Maria A. Aronova, Irina D. Pokrovskaya, Brian Storrie, Richard D. Leapman

**Affiliations:** 1 National Institute of Biomedical Imaging and Bioengineering, NIH, Bethesda, 20892 USA; 2 University of Maryland, College Park, 20740 USA; 3 University of Arkansas for Medical Sciences, Little Rock, 72205 USA

**Keywords:** Machine learning, Electron microscopy, Image processing

## Abstract

Biologists who use electron microscopy (EM) images to build nanoscale 3D models of whole cells and their organelles have historically been limited to small numbers of cells and cellular features due to constraints in imaging and analysis. This has been a major factor limiting insight into the complex variability of cellular environments. Modern EM can produce gigavoxel image volumes containing large numbers of cells, but accurate manual segmentation of image features is slow and limits the creation of cell models. Segmentation algorithms based on convolutional neural networks can process large volumes quickly, but achieving EM task accuracy goals often challenges current techniques. Here, we define *dense cellular segmentation* as a multiclass semantic segmentation task for modeling cells and large numbers of their organelles, and give an example in human blood platelets. We present an algorithm using novel hybrid 2D–3D segmentation networks to produce dense cellular segmentations with accuracy levels that outperform baseline methods and approach those of human annotators. To our knowledge, this work represents the first published approach to automating the creation of cell models with this level of structural detail.

## Introduction

Biomedical researchers use electron microscopy (EM) to image cells, organelles, and their constituents at the nanoscale. Today, the resulting image volumes can be gigavoxels in size or more, using hardware including the serial block-face scanning electron microscope (SBF-SEM)^1^, which employs automated serial sectioning techniques on block samples. This rapid growth in throughput challenges traditional image analytic workflows for EM, which rely on trained humans to identify salient image features. High-throughput EM offers to revolutionize structural biology by providing nanoscale structural detail across macroscopic tissue regions, but using these datasets in their entirety will be infeasibly time-consuming until analytic bottlenecks are addressed.

This paper develops the *dense cellular segmentation* method, a semantic segmentation task which classifies each voxel in an image into categories from a detailed schema of cellular and subcellular structures. Cell biologists have used similar segmentations of cellular structures to provide rich 3D ultrastructural models yielding new insights into cellular processes^2,3^, but applying this method across entire SBF-SEM datasets requires automation. Modeling 30 platelet cells across 3 physical platelet samples^2^ required nine months’ work from two in-lab annotators and represented a small fraction of all imaged cells.

It is challenging to automate dense segmentation tasks for EM due to the image complexity of biological structures at the nanoscale. An image with little noise and high contrast between features may be accurately segmented with simple thresholding methods, while accurate segmentation of images with multiscale features, noise, and textural content remains an open problem for many biomedical applications. Solving such segmentation problems algorithmically is one of many tasks in applied computer vision that has received increased interest in the past decade, as advances in deep neural network construction and training have driven significant computer vision performance improvements. Natural image-based applications of image segmentation have received enormous attention, with major companies and research institutions creating sophisticated trained neural networks in the pursuit of solutions to problems of economic importance^4–8^.

Work in biomedical imaging has been comparatively modest, but there nevertheless are thriving research communities working on problems in medical computed tomography (CT)^9,10^ and microscopy. A seminal contribution from this area was the U-Net^11^, which spawned numerous encoder-decoder variants demonstrating architectural improvements^12,13^ and helped popularize the encoder-decoder motif for segmentation problems in biomedical imaging. An important difference between biomedical and natural imaging is the ubiquity of volumetric imaging methods, including the SBF-SEM studied in this paper. These methods have spurred developments in volumetric segmentation, including 2D techniques applied to orthogonal slices of a 3D volume^14^, fully-3D segmentation^15–19^, as well as hybrid architectures that incorporate both 2D and 3D spatial processing^17,20,21^. Here, we have adapted existing 2D DeeplabV3^6^, 3D DeepVess^19^, and 2D and 3D U-Net architectures to our segmentation task as a baseline for our new results.

We find that for our application, hybrid 2D–3D networks work best. Building on previous work in this direction, we introduce a new 3D biomedical segmentation algorithm based on ensembles of neural networks with separated 2D and 3D convolutional modules and output heads. We show that our algorithm outperforms baselines in intersection-over-union (IoU) metrics, does a better job of maintaining boundaries between adjacent cellular structures, approaches the image quality of our human annotators, and closely matches human performance on a downstream biological analysis task. We use the algorithm to segment a billion-voxel block sample in an hour on a single NVIDIA GTX 1080 GPU, demonstrating a segmentation capability that is infeasible without automation and is accessible to commodity computing tools.

## Methods

SBF-SEM image volumes were obtained from identically-prepared platelet samples from two humans. Lab members manually segmented portions of each volume into seven classes to analyze the structure of the platelets. The labels were used for the supervised training of candidate network architectures, as well as baseline comparisons. We trained multiple instances of candidate architectures, each with different random initializations. The best-performing instances were ensembled together to produce the final segmentation algorithms used in this paper.

### Data collection

This study used datasets prepared from two human platelet samples as part of a collaborative effort between the National Institute of Biomedical Imaging and Bioengineering (NIBIB), NIH and the University of Arkansas for Medical Sciences. All human blood draws were approved by the University of Arkansas for Medical Sciences’ Institutional Review Board in accordance with national and international guidelines. All donors were informed of possible risks and signed an informed consent form. The platelet samples were imaged using a Zeiss Sigma 3View SBF-SEM. The Subject 1 dataset is a (*z*, *y*, *x*) $250\times 2000\times 2000$ voxel image with a lateral resolution in the $y-x$ plane of $10\,\text {nm}$ and an axial resolution along the *z*-axis of $50\,\text {nm}$, from a sample volume with dimensions $12.5\times 20\times 20\mu \text {m}^3$. The Subject 2 dataset is a $239\times 2000\times 2000$ voxel image produced by the same imaging protocol with the same lateral and axial resolutions.

We assembled labeled datasets from manually-segmented regions of the platelet image volumes. Lab members created tool-assisted manual segmentations using Amira^22^. Ground-truth labels for the training, evaluation, and test datasets were repeatedly reviewed by subject experts and corrected until accuracy standards were met, a slow feedback process that is necessary to produce high-quality labels. The Annotator 1 and Annotator 2 labels were created in a single pass by lab members without going through a review process from subject experts. As a result, the Annotator 1 and Annotator 2 labels are less accurate, but also much faster to produce. We use the high-quality ground-truth labels to train and validate all networks in this paper, but also compare algorithms against the unreviewed Annotator 1 and 2 labels as an additional measure of performance.

**Figure 1:**
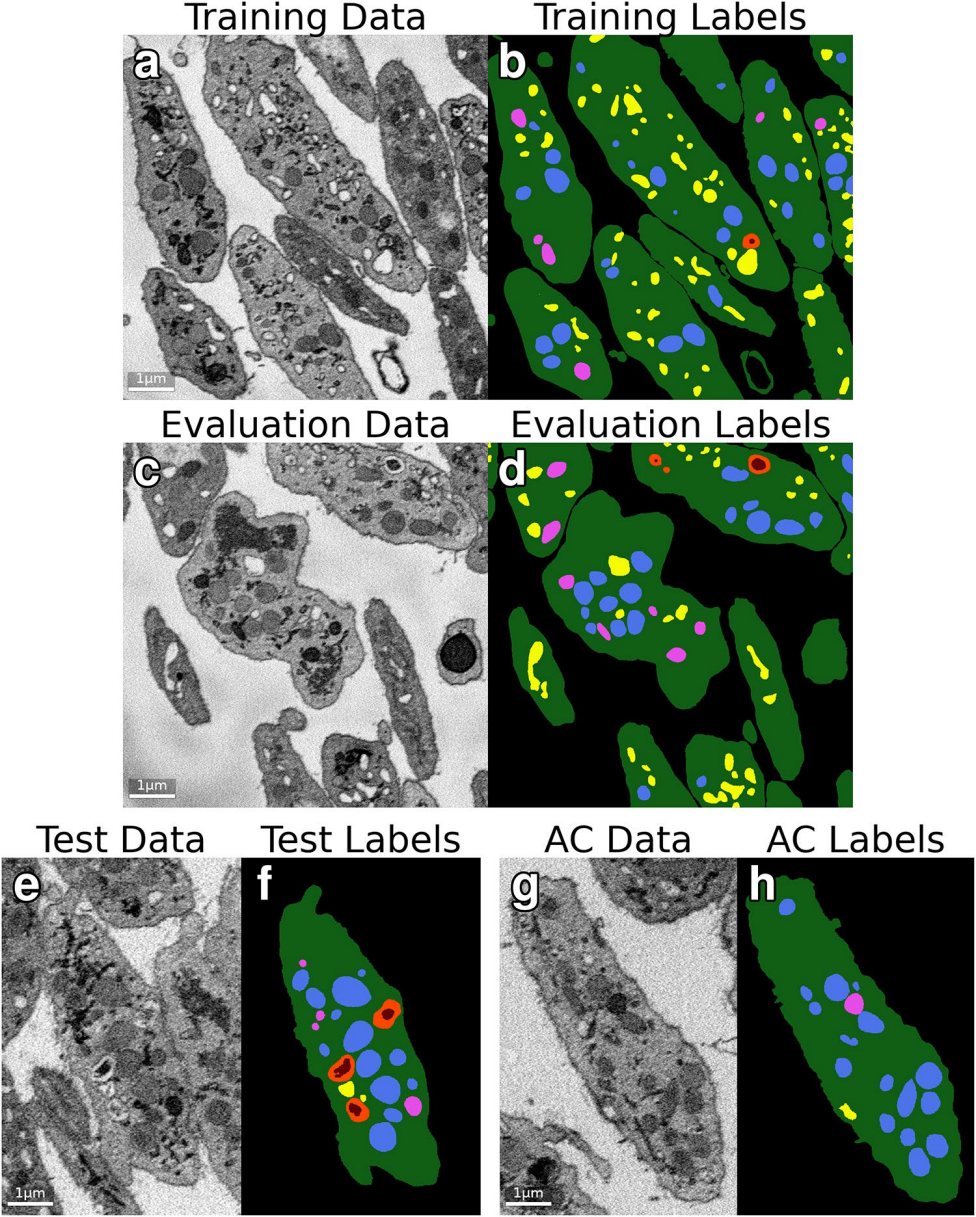
Dataset visualization. Sample $y-x$ orthoslices of the datasets used in this study. (**a**, **b**) One of the 50 training image data and label orthoslices. (**c**, **d**) One of the 24 evaluation image data and label orthoslices. (**e**, **f**) One of the 121 test image data and label orthoslices. (**g**, **h**) One of the 110 annotator comparison (AC) image data and label orthoslices.

The training image was a $50\times 800\times 800$ subvolume of the Subject 1 dataset spanning the region $81\le z\le 130$, $1073\le y\le 1872$, $620\le x\le 1419$ in 0-indexed notation. The evaluation image was a $24\times 800\times 800$ subvolume of the Subject 1 dataset spanning the region $100\le z\le 123$, $200\le y\le 999$, $620\le x\le 1419$. The test image was a $121\times 609\times 400$ subvolume of the Subject 2 dataset spanning the region $0\le z\le 120$, $460\le y\le 1068$, $308\le x\le 707$. The annotator comparison image was a $110\times 602\times 509$ subvolume of the Subject 2 dataset spanning the region $116\le z\le 225$, $638\le y\le 1239$, $966\le x\le 1474$. The training and evaluation labels covered the entirety of their respective images, while the test and annotator comparison labels covered a single cell contained within their image volumes. The labeling schema divides image content into seven classes: background (0), cell (1), mitochondrion (2), canalicular channel (3), alpha granule (4), dense granule (5), and dense granule core (6). Voxels labeled as the cell class include cytoplasm as well as organelles not accounted for in the labeling schema. Figure 1 shows sample images of the datasets and ground truth labels.

### Neural architectures and ensembling

The Subject 1 and Subject 2 datasets were binned by 2 in *x* and *y*, and aligned. For each of the training, evaluation, and testing procedures, the respective image subvolumes were normalized to have mean 0 and standard deviation 1 before further processing.

**Figure 2:**
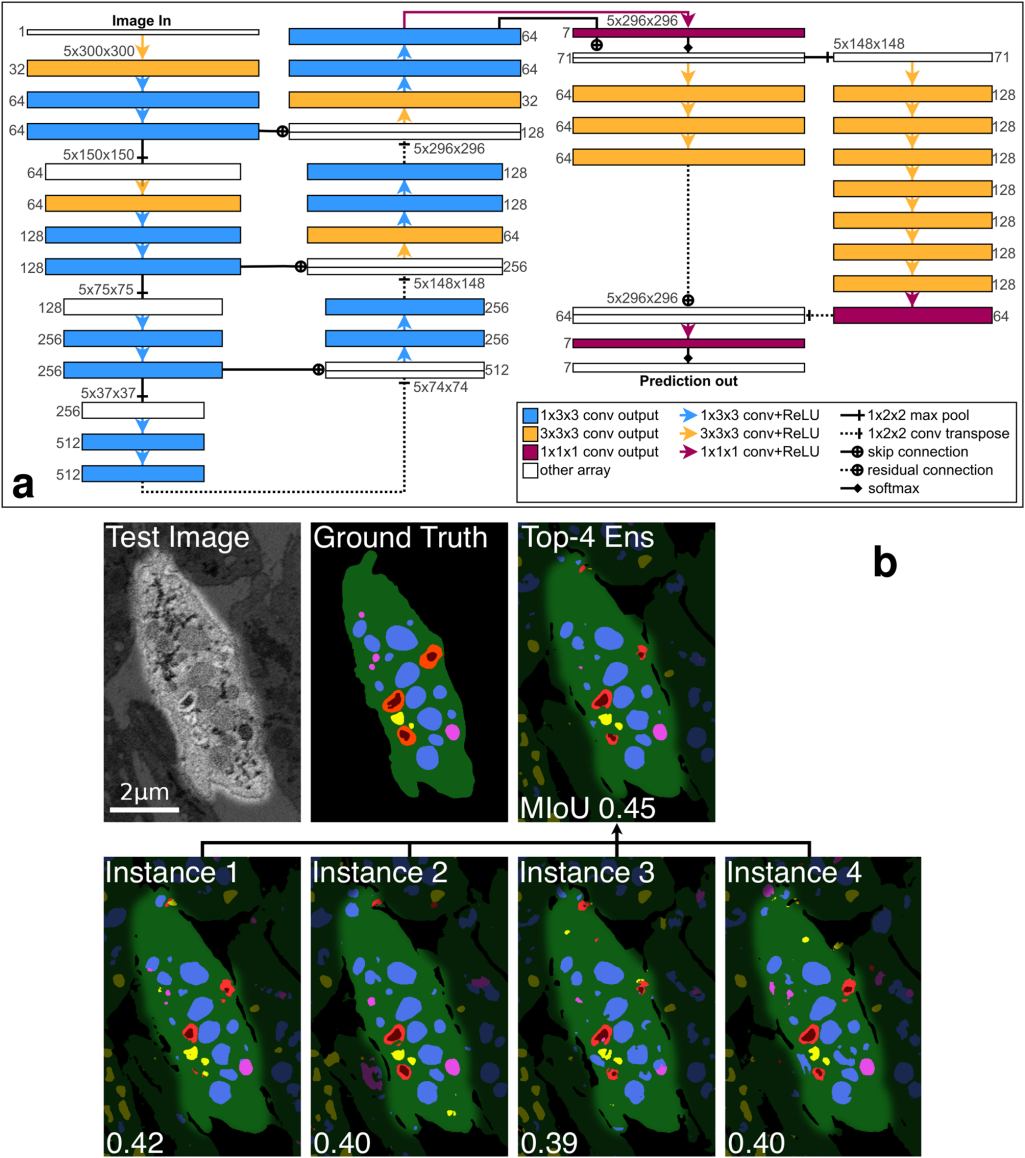
Methods. (**a**) Diagram of the 2D–3D + 3 $\times$ 3 $\times$ 3 network architecture, the best design tested in this paper. A 1-channel 3D image is passed through the network to produce a 7-channel output prediction of per-voxel probability distributions over the 7 label classes. Boxes represent multidimensional arrays, and arrows represent operations between them. Number triplets along box tops are array spatial axis sizes in (*z*, *y*, *x*) order. Numbers along box sides are array channel axis sizes. (**b**) Illustration of initialization-dependent performance of trained segmentation networks, and exploiting it for ensembling. An image of the test cell and ground truth labels are compared with segmentations of the best 4 trained 2D–3D + 3 $\times$ 3 $\times$ 3 network instances and an ensemble formed from them. The ensemble improves ${\rm{MIoU}}^{(org)}$ by 7.1% over the best single network.

The highest-performing network architecture in this paper, 2D–3D + 3 × 3 × 3, is a composition of a 2D U-net-style encoder-decoder and 3D convolutional spatial pyramid, with additional 3 × 3 × 3 convolutions at the beginning of convolution blocks in the 2D encoder-decoder. All convolutions are zero-padded to preserve array shape throughout the network, allowing deep architectures to operate on data windows with small *z*-dimension. A ReLU activation follows each convolution. All convolution and transposed convolutions use bias terms. The architecture is fully specified as a diagram in Fig. 2. Additionally, several baseline comparison networks and three 2D–3D + 3 × 3 × 3 ablation networks were also tested in this paper and are described in the Validation and Performance Metrics section.

To build a 2D–3D network, one can adapt a 2D U-net-style encoder-decoder module to work on 3D data by recasting 2D 3 × 3 convolutions as 1 × 3 × 3 convolutions, and 2D 2 × 2 max-pooling and transposed convolution layers as 1 × 2 × 2 equivalents. In this way, a 3D input volume can be processed in a single computation graph as a sequence of independent 2D regions in a 2D module and as a contiguous 3D region in a 3D module, and the 2D and 3D modules can be jointly trained end-to-end. This formulation also allows for seamless combination of batched-2D and 3D operations within a module, demonstrated in the 2D–3D + 3 × 3 × 3 architecture as 2D convolution block-initial 3 × 3 × 3 convolutions. Intermediate 2D class predictions ${\hat{x}}_{2D}$ are formed from the 2D module output, and the 2D output and class predictions are concatenated along the feature axis to form an input to a 3D spatial pyramid module. The 3D module applies a 1 × 2 × 2 max pool to its input to form a two-level spatial pyramid with scales 0 (input) and 1 (pooled). The pyramid elements separately pass through 3D convolution blocks, and the scale 1 block output is upsampled and added to the scale 0 block output with a residual connection to form the module output. 3D class predictions ${\hat{x}}_{3D}$ are formed from the 3D module output, and the final segmentation output ${\hat{\ell }}$ of the algorithm is a voxelwise argmax of the 3D class predictions. To build a 2D–3D + 3 × 3 × 3 network, we inserted 3 × 3 × 3 convolution layers at the beginning of the first two convolution blocks in the 2D encoder and the last two convolution blocks in the 2D decoder.

Given a collection of networks’ 3D class predictions, one can form an ensemble prediction by computing a voxelwise average of the predictions and computing a segmentation from that. Ensembling high-quality but non-identical predictions can produce better predictions^23^, and there is reason to think that more sophisticated ensembles could be constructed from collections of diverse neural architectures^24^, but in this paper we use a simple source of differing predictions to boost performance: ensembles of identical architectures trained from different random initializations. The sources of randomness in the training procedure are examined more thoroughly in the Validation and Performance Metrics section, but in our experiments this variation produced a small number of high-performing network instances per architecture with partially-uncorrelated errors.

### Network training

We consider a network predicting 7 classes $C=\{0, \ldots , 6\}$ for each voxel in a shape-$(o_z, o_x, o_y)$ data window $\Omega$ containing $N=o_zo_xo_y$ voxels $\{v_i\}_{i=1}^N$. The ground-truth segmentation of this region is a shape-$(o_z, o_x, o_y)$ array $\ell$ such that $\ell (v)\in C$ is the ground-truth label for voxel *v*. A network output prediction is a shape-$(7, o_z, o_x, o_y)$ array $\hat{x}$ such that $x_v\triangleq \hat{x}(:, v)$ is a probability distribution over possible class labels for voxel *v*. The corresponding segmentation ${\hat{\ell }}$ is the per-voxel $\arg \max$ of $\hat{x}$. Inversely, from $\ell$ one may construct a shape-$(7, o_z, o_x, o_y)$ per-voxel probability distribution *x* such that $x_v(i)=1$ if $i=\ell (v)$ and 0 if not, which is useful during training. All networks used a minibatch size of 1, so the minibatch axis is omitted from each of the array shape descriptions in this paper. Array shapes are given in (*C*, *Z*, *Y*, *X*) order, where *C* is the array size along the channel axis.

We trained our networks as a series of experiments, with each experiment training and evaluating 1 or more instances of a fixed network architecture. Instances within an experiment varied only in the random number generator (RNG) seed used to control trainable variable initialization and training data presentation order. In addition to the main 2D–3D + 3 × 3 × 3 architecture, there were three ablation experiments - No 3 × 3 × 3 Convs, No Multi-Loss, No 3D Pyramid - and five baseline experiments - Original U-Net, 3D U-Net Thin, 3D U-Net Thick, Deeplab + DRN, and Deeplab + ResNet101. Instances were trained and ranked by evaluation dataset MIoU. Experiments tracked evaluation MIoU for each instance at each evaluation point throughout training, and saved the final weight checkpoint as well as the checkpoint with highest evaluation MIoU. In this work we report evaluation MIoU checkpoints for each instance. The 2D–3D + 3 × 3 × 3 experiment and its ablations trained 26 instances for 40 epochs with minibatch size 1 (33k steps). The Original U-Net experiment trained 500 instances for 100 epochs with minibatch size 1 (180k steps). The 3D U-Net Thin experiment trained 26 instances for 100 epochs with minibatch size 1 (29k steps), and the 3D U-Net Thick experiment trained 26 instances for 100 epochs with minibatch size 1 (30k steps). The Deeplab + DRN and Deeplab + ResNet101 experiments trained 1 instance each for 200 epochs with minibatch size 4 (360k steps). Due to poor performance and slow training times of the Deeplab models, we deemed it unnecessary to train further instances. Networks were trained on NVIDIA GTX 1080 and NVIDIA Tesla P100 GPUs.

This subsection details the training of the 2D–3D + 3 × 3 × 3 network. Baseline and ablation networks were trained identically except as noted in Validation and Performance Metrics. All trainable variables were initialized from Xavier uniform distributions. Each instance was trained for 40 epochs on shape-(1, 5, 300, 300) windows extracted from the training volume, and output a shape-(7, 5, 296, 296) class prediction array. The number of windows in each epoch was determined by a window spacing parameter which determined the distance along each axis between the top-back-left corners of each window, here (2, 100, 100), resulting in 828 windows per epoch. An early stopping criterion halted the training of any network that failed to reach an MIoU of 0.3 after 10 epochs.

Networks were trained using a regularized, weighted sum of cross-entropy functions. The network has a set $\Theta$ trainable variables divided into four subsets: $\Theta _{2D}$ for variables in the 2D encoder-decoder module, $\Theta _{3D}$ for variables in the 3D spatial pyramid module, the single 1 × 1 × 1 convolution variable $\{\theta _{2DP}\}$ which produces intermediate 2D class predictions $\hat{x}_{2D}$ from the encoder-decoder’s 64 output features, and the single 1 × 1 × 1 convolution variable $\{\theta _{3DP}\}$ which produces the final 3D class predictions $\hat{x}_{3D}$ from the spatial pyramid’s 64 output features. The loss function comparing predictions against ground-truth labels is

1 $$\begin{aligned} L(x, \hat{x}_{3D}, \hat{x}_{2D}; \Theta ) =&\frac{1}{N}\sum _{i=1}^N\left[ \mathcal {W}\otimes \mathcal {H}\left( x, \hat{x}_{3D}\right) \right] _i + \frac{c_{2D}}{N}\sum _{i=1}^N\left[ \mathcal {W}\otimes \mathcal {H}\left( x, \hat{x}_{2D}\right) \right] \nonumber \\&+ \lambda _{2D}\sum _{\theta \in \Theta _{2D}}\Vert \theta \Vert _2^2 + \lambda _{3D}\sum _{\theta \in \Theta _{3D}}\Vert \theta \Vert _2^2+\lambda _{P}\left( \Vert \theta _{2DP}\Vert _2^2+\Vert \theta _{3DP}\Vert 
_2^2\right) , \end{aligned}$$

where $\lambda _{2D}=1\times 10^{-4.7}$ and $\lambda _{3D}=1\times 10^{-5}$ are $L^2$ regularization hyperparameters for the variables in $\Theta _{2D}$ and $\Theta _{3D}$, $\lambda _P=1\times 10^{-9}$ is an $L^2$ regularization hyperparameter for the predictor variables $\theta _{2DP}$ and $\theta _{3DP}$, and $c_{2D}=0.33$ is a constant that weights the importance of the intermediate 2D class predictions in the loss function. $\mathcal {H}(x,\hat{x})$ is the voxelwise cross-entropy function, i.e.,

$$\begin{aligned} \mathcal {H}(x,\hat{x})_v\triangleq H(x_v, \hat{x}_v)\triangleq -\sum _{j=1}^7x_v(j)\log \left[ \hat{x}_v(j)\right] =-x_v(\ell _v)\log \left[ \hat{x}_v(\ell _v)\right] . \end{aligned}$$

$\mathcal {W}$ is a shape-(5, 296, 296) array of weights; its Kronecker product with $\mathcal {H}$ produces a relative weighting of the cross-entropy error per voxel. This weighting strategy is based generally on the approach in Ronneberger et al.^11^:

$$\begin{aligned} \mathcal {W}\triangleq w+\mathcal {W}_{cb}+\mathcal {W}_{ep}. \end{aligned}$$

The initial $w=0.01$ is a constant that sets a floor for the minimum weight value, $\mathcal {W}_{cb}$ is a class-balancing term such that $\mathcal {W}_{cb,i}\propto 1/N_i$, where $N_i$ is the number of occurrences in the training data of $\ell _i$, rescaled so that $\max {\mathcal {W}_{cb}}=1$. $\mathcal {W}_{ep}$ is an edge-preserving term that upweights voxels near boundaries between image objects and within small 2D cross-sections. In Ronneberger et al.^11^ this is computed using morphological operations. We used a sum of scaled, thresholded diffusion operations to approximate this strategy in a manner that requires no morphological information. $\mathcal {W}_{ep}$ is built up as a rectified sum of four terms:

$$\begin{aligned} \mathcal {W}_{ep}\triangleq R_{\alpha }\left( \mathcal {W}_{bkgd\rightarrow cell} + \mathcal {W}_{cell\rightarrow bkgd} + \mathcal {W}_{cell\rightarrow org} + \mathcal {W}_{org\rightarrow cell}\right) , \end{aligned}$$

where $R_{\alpha }(W)=\mathrm {ReLU}(W-\alpha )\cdot \frac{\max {W}}{\max {W}-a}$. For each term, we choose two disjoint subsets $C_{source}$ and $C_{target}$ of the classes *C*. Let $\ell _{source}$ be the binary image such that $\ell _{source}(v)=1$ if $\ell (v)\in C_{source}$ and $\ell _{source}(v)=0$ otherwise. Define

$$M_{source}(c, \sigma )\triangleq c\cdot \ell _{source} * k_{\sigma },$$

where $*$ denotes convolution and $k_{\sigma }$ is a Gaussian diffusion kernel with standard deviation $\sigma$. Then, $\mathcal {W}_{source\rightarrow target}(v)=\mathcal {M}_{source}(v)$ if $\ell (v)\in C_{target}$, and is 0 otherwise. The terms in the $\mathcal {W}_{ep}$ array used in this paper were computed using class subsets $bkgd=\{0\}$, $cell=\{1\}$, and $org=\{2, 3, 4, 5, 6\}$, $\alpha =0.25$, $c=0.882$, and $\sigma =6$. The error weighting array used in this paper and the code used to generate it are available with the rest of the platelet dataset at https://leapmanlab.github.io/dense-cell. See Figure S6 for a visualization of the error weighting array. $\mathcal {W}_{cb}$ is calculated all at once across the entire 3D training volume, while $\mathcal {W}_{ep}$ is calculated independently per each 2D *z*-slice of the training volume.

We employed data augmentation to partially compensate for the limited available training data. Augmentations were random reflections along each axis, random shifts in brightness ($\pm 12\%$) and contrast ($\pm 20\%$), and elastic deformation as in Ronneberger et al.^11^. For elastic deformation, each 800x800 $x-y$ plane in the shape-(50, 800, 800) training data and label arrays was displaced according to a shape-(800, 800, 2) array of 2D random pixel displacement vectors, generated by bilinearly upsampling a shape-(20, 20, 2) array of independent and identically distributed Gaussian random variables with mean 20 and standard deviation 0.6. During each epoch of training, a single displacement map was created and applied to the entire training volume before creating the epoch’s batch of input and output windows. Training used the ADAM optimizer with learning rate $1\times 10^{-3}$, $\beta _1=1-1\times 10^{-1.5}$, $\beta _2=1-1\times 10^{-2.1}$, and $\epsilon =1\times 10^{-7}$. Training also used learning rate decay with an exponential decay rate of 0.75 every $1\times 10^{3.4}$ training iterations.

### Validation and performance metrics

The performance metric used in this work is mean intersection-over-union (MIoU) between ground-truth image segmentation $\ell$’s 7 labeled sets $\{L_j=v\in \Omega \,|\,\ell (v)=j\}_{j\in C}$ and predicted segmentation’s ${\hat{\ell }}$ labeled sets $\{{\hat{L}}_j=v\in \Omega \,|\,{\hat{\ell }}(v)=j\}_{j\in C}$. Given two sets *A* and *B*, $\mathrm {IoU}(A,B)=\frac{\left| A\cap B\right| }{\left| A\cup B\right| }.$ Then for segmentations $\ell$ and ${\hat{\ell }}$ with their corresponding labeled sets over the 7 semantic classes, ${\rm{MIoU}}(\ell , {\hat{\ell }})=\frac{1}{7}\sum _{j\in C}{\mathrm{IoU}}(L_j, {\hat{L}}_j).$ More generally, for a subset of labels $D\subseteq C$, one can compute the MIoU over *D*, or ${\rm{MIoU}}^{(D)}$, as

$$\begin{aligned} {\rm{MIoU}}^{(D)}(\ell , {\hat{\ell }})=\frac{1}{|D|}\sum _{j\in D}{\mathrm{IoU}}(L_j, {\hat{L}}_j). \end{aligned}$$

Note that this definition weights the IoU scores for each class equally, regardless of the number of examples of each class in the dataset. One may choose to use a class frequency-weighted MIoU instead to reflect this class imbalance, but we choose to use an unweighted MIoU to emphasize performance on rarer classes.

Here we are concerned with MIoUs over two sets of labels: ${\rm{MIoU}}^{(all)}$ over the set of all 7 class labels, and ${\rm{MIoU}}^{(org)}$ over the set of 5 organelle labels 2-7. Our network validation metrics were ${\rm{MIoU}}^{(all)}$ and ${\rm{MIoU}}^{(org)}$ on the evaluation dataset, and ${\rm{MIoU}}^{(org)}$ on the test dataset. Test data uses ${\rm{MIoU}}^{(org)}$ because the labeled region is a single cell among several unlabeled ones, and restricting validation to the labeled region invalidates MIoU statistics for the background and cell classes (0 and 1). We include evaluation ${\rm{MIoU}}^{(org)}$ to quantify how performance drops between a region taken from the physical sample used to generate the training data, and a new physical sample of the same tissue system.

Using this procedure, the performance of the 2D–3D + 3 × 3 × 3 network was compared against three ablations and five baseline networks. The three ablations each tested one of three features that distinguish the 2D–3D + 3 × 3 × 3 network in this paper from similar baselines. The first, 2D + 3 × 3 × 3 No 3 × 3 × 3 Convs, replaces the 3 × 3 × 3 convolutions in the net’s encoder-decoder module with 1 × 3 × 3 convolutions that are otherwise identical. With this ablation, the network’s encoder-decoder loses any fully-3D layers. The second, 2D + 3 × 3 × 3 No Multi-Loss, modifies the loss function in Eq. (1) by removing the term involving $\hat{x}_{2D}$ but otherwise leaving the architecture and training procedure unchanged. This ablation tests whether it is important to have auxiliary accuracy loss terms during training. The third ablation, 2D–3D + 3 × 3 × 3 No 3D Pyramid, removes the 3D spatial pyramid module and 3D class predictor module from the network architecture, so that $\hat{x}_{2D}$ is the network’s output. Correspondingly, the loss term involving $\hat{x}_{3D}$ is removed from Eq. (1).

We implemented five baseline networks by adapting common models in the literature to our platelet segmentation problem. Three of these were 2D - The original U-Net^11^ as well as two Deeplab variants^6,7^ using a deep residual network (DRN) backbone and a ResNet101 backbone^25^, minimally modified to output 7 class predictions. The original U-Net used (572, 572) input windows and (388, 388) output windows, while the Deeplab variants used (572, 572) input and output windows. The two 3D networks were fully-3D U-Net variants adapted on the 3D U-Net in (Çiçek et al., 2016)^26^ - 3D U-Net Thin and 3D U-Net Thick. The variants used same-padding, had three convolutions per convolution block, and two pooling operations in the encoder for convolution blocks at three spatial scales. The 3D U-Net Thin network used (5, 300, 300) input windows and (5, 296, 296) output windows, and pooling and upsampling operations did not affect the *z* spatial axis. The 3D U-Net Thick network used (16, 180, 180) input windows and (16, 180, 180) output windows, and pooled and upsampled along all three spatial axes.

To determine whether one architecture is superior to another, trained instances are compared with each other. However, sources of randomness in the training process induce a distribution of final performance metric scores across trained instances of an architecture, so that a single sample per architecture may be insufficient to determine which is better. While expensive, a collection of instances can be trained and evaluated to empirically approximate the performance distribution for each architecture. In this way, better inferences may be made about architecture design choices. Figure S5 shows the empirical performance distributions for the 26 trials of the 2D–3D + 3 × 3 × 3 architecture and its three ablations, as well as the 26 trials of the 3D U-Net and 500 trials of the 2D Original U-Net.

In addition to the multiclass baselines, we chose to also evaluate a CDeep3M plug-and-play system^27^ that can be spun up on Amazon Web Services (AWS) for binary segmentation problems. In a similar vein to our work and others’, they use an ensemble of convolutional neural networks to perform binary segmentation tasks. This differs from the multiclass segmentation problems that we address, but their polished workflow makes it easy to replicate and train on new data. We therefore decided to evaluate CDeep3M on a comparable binary segmentation task with our data, wherein all non-background classes were grouped together into a single cell class. Using the AWS stack provided on the project GitHub page (https://github.com/CRBS/cdeep3m), we trained the networks used in their 3D segmentation ensemble for 30000 iterations on our training dataset, using all other default hyperparameters. Training took approximately 96 hours on an Amazon EC2 instance with an NVIDIA P100 GPU card.

After training completed, we ran the CDeep3M 3D ensemble’s prediction tool on our evaluation dataset, and compared it with a binarized version of our best algorithm’s segmentation of the evaluation dataset. We binarized our algorithm’s segmentation the same way we binarized our ground truth labels, by mapping together all the non-background segmented classes. The CDeep3M algorithm, however, produces a single per-voxel probability map that indicates the probability each voxel belongs to a cell region. To compute a segmentation from the probability map, a cutoff threshold *t* must be specified - a segmentation with threshold *t* assigns the cell class to all voxels with probability greater than *t*, and background to all others. We computed MIoU scores for our lab’s (LCIMB) segmentation, as well as CDeep3M segmentations with thresholds in $\{0, 0.1, 0.2, 0.3, 0.4, 0.5, 0.6, 0.7, 0.8, 0.9, 1\}$.

A final point of comparison was drawn between the top algorithm’s performance and the initial work of laboratory scientific image annotators, Annotator 1 and Annotator 2. The three sources each labeled the annotator comparison region from the Subject 2 platelet sample. Pairwise ${\rm{MIoU}}^{(org)}$ scores and organelle confusion matrices were calculated to compare the level of disagreement between two human labelings and between humans and the algorithm. We also computed organelle volume fractions for each segmentation to compare performance in segmentation applications to downstream analysis tasks. The cell volume fraction of an organelle is equal to the summed voxels of all organelles in a cell, divided by the cell’s volume. To compute this quantity for each organelle, the number of voxels for each organelle label is divided by the number of voxels in the cell. For the algorithmic result, since the semantic segmentation map does not distinguish between separate cells in the field of view, a mask for the single annotator comparison dataset cell was approximated as all non-background-labeled voxels in a small region around the Annotator 1 cell mask.

## Results

Inspired by existing work on combining 2D and 3D computations for volumetric data analysis^20,21^ we experiment with combinations of 2D and 3D neural modules to trade off between computational efficiency and spatial context. The highest-performing network architecture in this paper, 2D–3D + 3 × 3 × 3, is a composition of a 2D U-Net-style encoder-decoder and 3D convolutional spatial pyramid, with additional 3 × 3 × 3 convolutions at the beginning of convolution blocks in the encoder-decoder. We use same-padded convolution operations throughout, so that 3D operations can be used on anisotropic data windows with small size along the *z* axis.

Our best algorithms as defined by MIoU score are ensembles that average the per-voxel class probability distributions across several networks. The ensembled networks are identical architectures trained from different random weight initializations. When describing segmentation algorithms, we use Top-*k* to indicate an ensemble of the best *k* instances of an architecture. Figure 2 details our best network architecture and illustrates the ensembling process.

**Figure 3:**
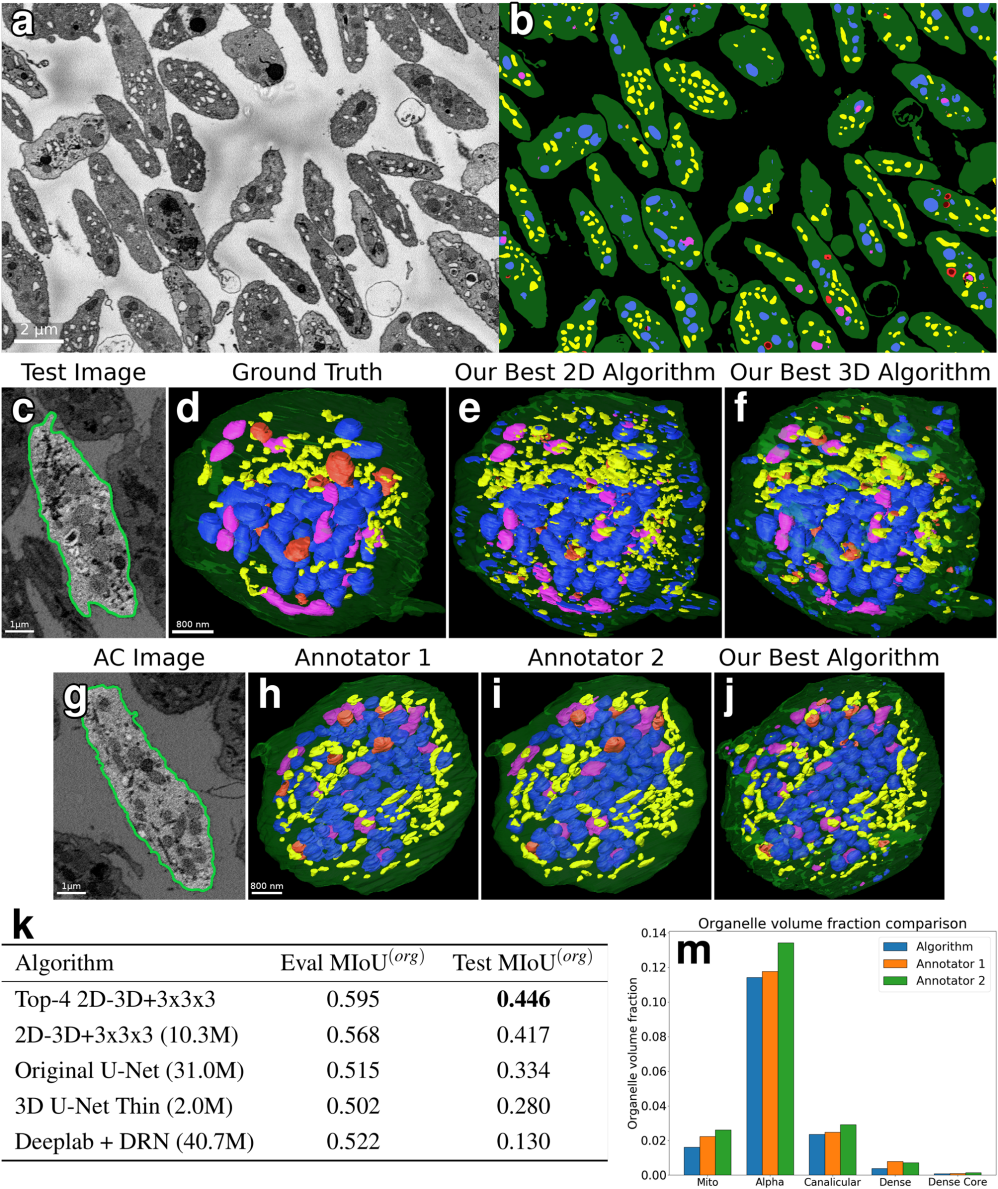
Results. (**a**, **b**) Orthoslice of Subject 1 image and segmentation. (**c**) Test dataset orthoslice, segmented cell highlighted. (**d**, **f**) Comparison between ground truth segmentation of test cell and our best 2D and 3D algorithms. (**g**) Annotator comparison (AC) dataset orthoslice, segmented cell highlighted. (**h**, **j**) Annotator comparison cell segmentations, comparing the two human annotators and our best (3D) algorithm. (**k**) Summarized comparison of mean intersection-over-union across organelle classes (${\rm{MIoU}}^{(org)}$) on test and evaluation datasets for segmentation algorithms. For full results, see Table S1. (**m**) Comparison of organelle volume fractions between two human annotators and our best algorithm, computed from annotator comparison cell segmentations.

For the main experiment of this study, we train baseline architectures from the literature, our new architecture, and ablations of the new architecture on a dense cellular segmentation task by supervised learning from the training dataset. We compare single-network and ensemble segmentation performance on the evaluation and test datasets. We conclude that our algorithm outperforms baselines, the differentiating features of our final best architecture are responsible for the performance differences, and that multi-instance ensembles significantly improve performance over single networks. The results of this experiment are shown in Fig. 3. We consider test performance to be the best indicator of an algorithm’s performance as it shows its ability to generalize across different samples. Figure 3 row 2 compares visualizations of the best 3D segmentation algorithms with ground-truth labels and image data for the test dataset. Figure 3 row 4 highlights the most notable performance results, and more performance statistics can be found in Table S1. Additional 3D renderings comparing manual and algorithmic performance are shown in Figures S1 and S3, and a 2D comparison of segmentations of the evaluation dataset by all networks tested in this paper is shown in Figure S2.

We also compare our best algorithm against the segmentations of scientific image annotators, Annotator 1 and Annotator 2, who are laboratory staff trained on annotation tasks but are not biological domain experts. These initial segmentations are currently the first step in producing high-quality dense cellular segmentations, and even before any corrections they require 1-2 work days per cell to create. Results are displayed in Fig. 3 row 3, with further details in Figures S3 and S4. Annotator 1, Annotator 2, and our algorithm each labeled the annotator comparison region from the Subject 2 platelet sample. We calculated ${\rm{MIoU}}^{(org)}$ scores pairwise from the three segmentations: 0.571 for Annotator 1 vs. Annotator 2, 0.497 for Annotator 1 vs. Algorithm, and 0.483 for Annotator 2 vs. Algorithm. The confusion matrices in Figure S4 further break down results by organelle class. The statistics indicate that our algorithm disagrees more with either annotator than the annotators do with each other, but none of the labels are consistent everywhere, reflecting the difficulty of dense cellular segmentation even for humans.

**Figure 4:**
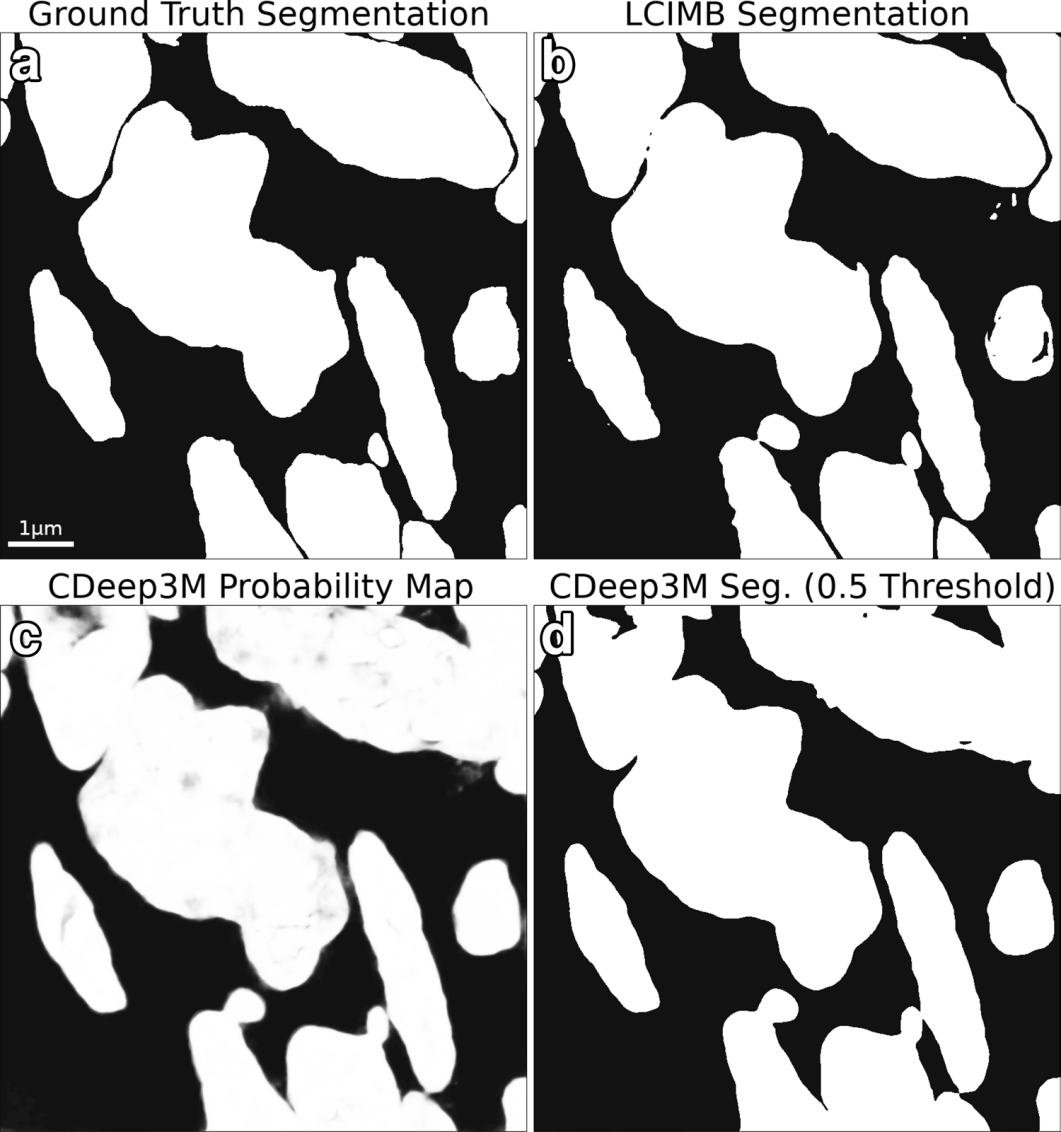
CDeep3M segmentation comparison. Comparison between the CDeep3M segmentation tool and our lab’s (LCIMB) best segmentation algorithm for a binary cell/non-cell segmentation problem on our evaluation dataset. (**a**) Orthoslice of the ground truth binary segmentation of the evaluation dataset. (**b**) Segmentation using our lab’s (LCIMB) best 3D ensemble. (**c**) Probability map produced by the CDeep3M ensemble after training on our data for 30000 iterations. The probability map is a per-voxel probability that the voxel belongs to a cell region, and it must be thresholded to produce a segmentation. (**d**) Segmentation from the CDeep3M ensemble with the best tested threshold of 0.5. This resulted in an MIoU of 0.935, compared to 0.946 for the LCIMB segmentation. In addition to a slight improvement in MIoU statistic, the LCIMB segmentation does a much better job of preserving boundaries between adjacent cells.

Our final direct segmentation evaluation was on the binary task whose results were compared with CDeep3M. The 0.4 and 0.5 thresholds both produced the highest MIoU score - 0.935. In contrast, the LCIMB segmentation had an MIoU of 0.946. Both algorithms generally did a good job of detecting cell material, but the LCIMB segmentation did a much better job of preserving boundaries between adjacent cells. The results can be seen in Fig. 4.

We are also interested in understanding how even imperfect segmentations may be useful for downstream analysis tasks. To this end, we computed organelle volume fractions for each organelle within the cell in the annotator comparison dataset. The cell volume fraction of an organelle is equal to the summed voxels of all organelles in a cell, divided by the cell’s volume. Biologists can correlate this information with other cell features to better understand variations in the makeup of cellular structures across large samples. The results in Fig. 3 row 4 show that our algorithm tended to underestimate volume fractions relative to the two annotators, but the difference between the algorithm and Annotator 1 is smaller than the difference between Annotator 1 and Annotator 2. The best 3D algorithm improves considerably over the best 2D algorithm. All algorithms detect small regions ignored by humans, but simple postprocessing with small region removal fails to significantly improve quality metrics.

## Discussion

We have argued here that dense semantic labeling of 3D EM images for biomedicine is an image analysis method with transformative potential for structural biology. We demonstrated that while challenges exist for both human and algorithmic labelers, automated methods are approaching the performance of trained humans, and we plan to integrate them into annotation software for greatly enhancing the productivity of humans segmenting large datasets. We have carefully evaluated adaptations of multiple common network architectures for our task, and demonstrated that a novel variant of 2D–3D fully convolutional network performs best. Without question, challenges remain for creating algorithms that are robust to the many types of variation present across research applications. However, SBF-SEM analysis problems are a fertile early ground for this computer vision research, as their large dataset sizes make the entire train-test-deploy cycle of supervised learning viable for accelerating analysis of even individual samples. We believe that the image in Fig. 3a,b showcases this best—after manually segmenting less than $1\%$ of the Subject 1 dataset, we were able to train a segmentation algorithm that produces a high-quality segmentation of the full dataset, a feat that would be impossible with anything short of an army of human annotators. While gains in accuracy will be realized with future developments, the procedure of training neural network ensembles on a manually annotated portion of a large SBF-SEM dataset is already becoming viable for making dense cellular segmentation a reality.

### Online content

Supplementary materials, source data, code, and reproducible examples are available online at https://leapmanlab.github.io/dense-cell.

## Supplementary Information

Supplementary Information.
