# Supplementary material for: Dense cellular segmentation for EM using 2D–3D neural network ensembles
Source: Sci Rep. 2021 Jan 28;11:2561. doi: 10.1038/s41598-021-81590-0 (PMC7844272; doi:10.1038/s41598-021-81590-0)
Supplement: Supplementary file 1 — Supplementary Information. [file 41598_2021_81590_MOESM1_ESM.pdf]

Dense cellular segmentation for EM using 2D-3D neural network ensembles

Matthew D. Guay[1,*], Zeyad A.S. Emam[1,2], Adam B. Anderson[1,2], Maria A. Aronova[1], Irina D. Pokrovskaya[3], Brian Storrie[3], and Richard D. Leapman[1]

[1]National Institute of Biomedical Imaging and Bioengineering, NIH, Bethesda, 20892, USA
[2]University of Maryland, College Park, 20740, USA
[3]University of Arkansas for Medical Sciences, Little Rock, 72205, USA
[*]matthew.guay@nih.gov

Supplementary Material

Additional segmentation visualizations

In addition to the renderings in the main paper, we produced 3D renderings of segmentation results for the evaluation dataset, as shown in Figure S1, showing results for all organelles together as well as separately for the ground-truth labels, as well as the best 2D and 3D segmentation algorithms. Similarly, Figure S3 shows renderings per each organelle class for Annotator 1, Annotator 2, and our best algorithm on the annotator comparison dataset. Finally, Figure S2 shows 2D images of segmentations for each of the 14 algorithms tested in this paper, which are also detailed in Table S1.

Ablation analysis and initialization-dependent performance

Our ablation analysis procedure, described in the **Validation and performance metrics** subsection of **Methods** confirms our conjectures about the importance of 3D context input to the network, and the importance of 3x3x3 convolutions over 1x3x3 convolutions for generalization performance. The latter do not capture correlations along the z spatial dimension, likely contributing to their poorer performance. Ablation analysis also indicates that removing either the multi-loss training setup or the 3D spatial pyramid module from the 2D-3D+3x3x3 architecture carries significant performance penalties. Removing either the 3x3x3 convolution layers or the 3D spatial pyramid on their own had a small effect on performance compared with removing the 2D loss term from the multi-loss objective function. Summary statistics demonstrating these results can be seen in Table S1(a), but these statistics only tell part of the story. Especially when effect sizes are small, looking at a single trained instance of each architecture may not be enough to determine relative performance between architecture candidates. To get a better idea of the effects of different architecture choices, we must deal with the initialization-dependent performance of these segmentation networks.

In Figure S5 we experiment with various weight initialization random seeds to determine the robustness of various

models to the weight initialization scheme. In order to determine whether one architecture choice is superior to another, the outputs of different trained networks are compared with each other. However, sources of randomness in the training process (initialization of trainable weights from a Xavier uniform distribution, and the random presentation order of training data elements) induce a distribution of final performance metric scores. These scores are random variables, and a single sample per architecture may be insufficient to determine which is better. By empirically approximating the distribution for each architecture, better inferences may be made about architecture design choices. For this figure, multiple instances of the same architecture (26 for 2D-3D and fully-3D nets, 500 for the U-Net) were trained under identical conditions, varying only random number generation seeds. The resulting distributions support the conclusions that 2D-3D networks outperform their 2D and fully-3D counterparts, as well as the conclusions drawn from the ablation studies. They also reveal a curious phenomenon that may be a topic for future study - the seemingly bimodal performance of 2D-3D architectures, wherein some fraction of trained instances perform markedly worse than others with an apparent performance gap between peaks. Whether this is a real phenomenon or an artifact of having an insufficient number of samples could be determined with a followup study.

DeepVess baseline comparison

In addition to the baseline models discussed in the main text, we have also tried using the DeepVess model from (Haft-Javaherian et al., 2019)[1] on our data. However, DeepVess performed poorly, and learned to assign a single class (background) to the entire output patch. We believe there may be two reasons behind DeepVess' poor performance: (1) Unlike U-Net and Deeplab networks, the DeepVess network is designed with very small input patches in mind; small patches do not contain enough context for the network to distinguish between objects. (2) DeepVess' last layer consists of a fully-connected operation with a single hidden layer containing 1024 neurons, therefore any attempt to input significantly larger patches would require increasing the number of neurons in the last layer, but fully-connected layers do not scale well and the network quickly outgrows GPU memory.

Segmentation 3D rendering videos

In addition to the 3D rendering images of segmentations displayed in figures in this work, we produced videos showing rotations of the renderings.

Evaluation dataset

Ground truth: `https://leapmanlab.github.io/dense-cell/vids/eval_gt.mp4`

Our best 3D ensemble: `https://leapmanlab.github.io/dense-cell/vids/eval_e-3d.mp4`

Our best 2D ensemble: https://leapmanlab.github.io/dense-cell/vids/eval_e-2d.mp4

Our best 3D network: https://leapmanlab.github.io/dense-cell/vids/eval_s-3d.mp4

Our best 2D network: https://leapmanlab.github.io/dense-cell/vids/eval_s-2d.mp4

Test dataset

Ground truth: https://leapmanlab.github.io/dense-cell/vids/test_gt.mp4

Our best 3D ensemble: https://leapmanlab.github.io/dense-cell/vids/test_e-3d.mp4

Our best 2D ensemble: https://leapmanlab.github.io/dense-cell/vids/test_e-2d.mp4

Our best 3D network: https://leapmanlab.github.io/dense-cell/vids/test_s-3d.mp4

Our best 2D network: https://leapmanlab.github.io/dense-cell/vids/test_s-2d.mp4

Annotator comparison dataset

Annotator 1: https://leapmanlab.github.io/dense-cell/vids/ac_ann1.mp4

Annotator 2: https://leapmanlab.github.io/dense-cell/vids/ac_ann2.mp4

Our best algorithm: https://leapmanlab.github.io/dense-cell/vids/ac_alg.mp4

Training demonstration videos

We trained a 2D-3D+3x3x3 network for 39744 iterations, recording the class prediction probability maps and segmentation that the network produced on the evaluation dataset every 92 iterations. We produced animations of the evolution of the network's prediction capabilities to demonstrate the learning process.

Probability maps video

The first video shows the evolution of the six non-background probability maps predicted by the network over the course of training. Each probability map is color-coded based on the corresponding structure color in the segmentation color scheme used throughout this paper - dark green for `cell`, magenta for `mitochondrion`, dark blue for `alpha granule`, yellow for `canalicular channel`, bright red for `dense granule`, and dark red for `dense granule core`.

Link: https://leapmanlab.github.io/dense-cell/vids/train_prob-maps.mp4

Segmentation video

The second video shows the evolution of the segmentation produced by the network over the course of training.

Link: https://leapmanlab.github.io/dense-cell/vids/train_seg.mp4

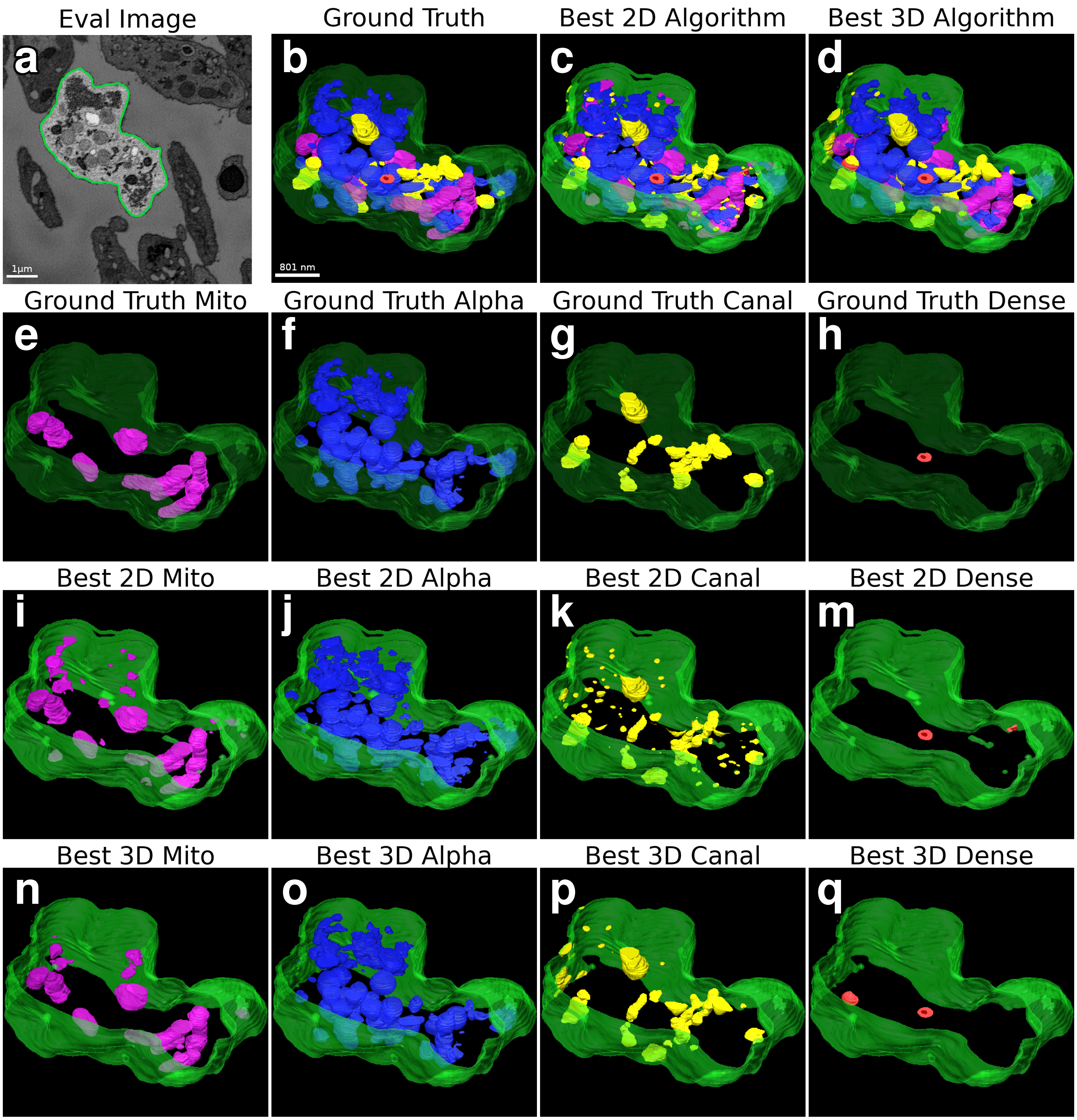

Figure S1. Evaluation dataset segmentation renderings. In each subfigure, "best" refers to our best 2D or 3D segmentation algorithm out of the ones we evaluated. (**a**) Orthoslice of the evaluation dataset with rendered cell highlighted. (**b**-**d**) Ground truth and our best 2D and 3D algorithm segmentations of the evaluation cell region showing all organelles. (**e**-**h**) Ground truth segmentations of individual organelles - from left to right: mitochondria (Mito), alpha granules (Alpha), canalicular channels (Canal), dense granules (Dense). (**i**-**m**) Our best 2D algorithm segmentations of individual organelles. (**n**-**q**) Our best 3D algorithm segmentations of individual organelles.

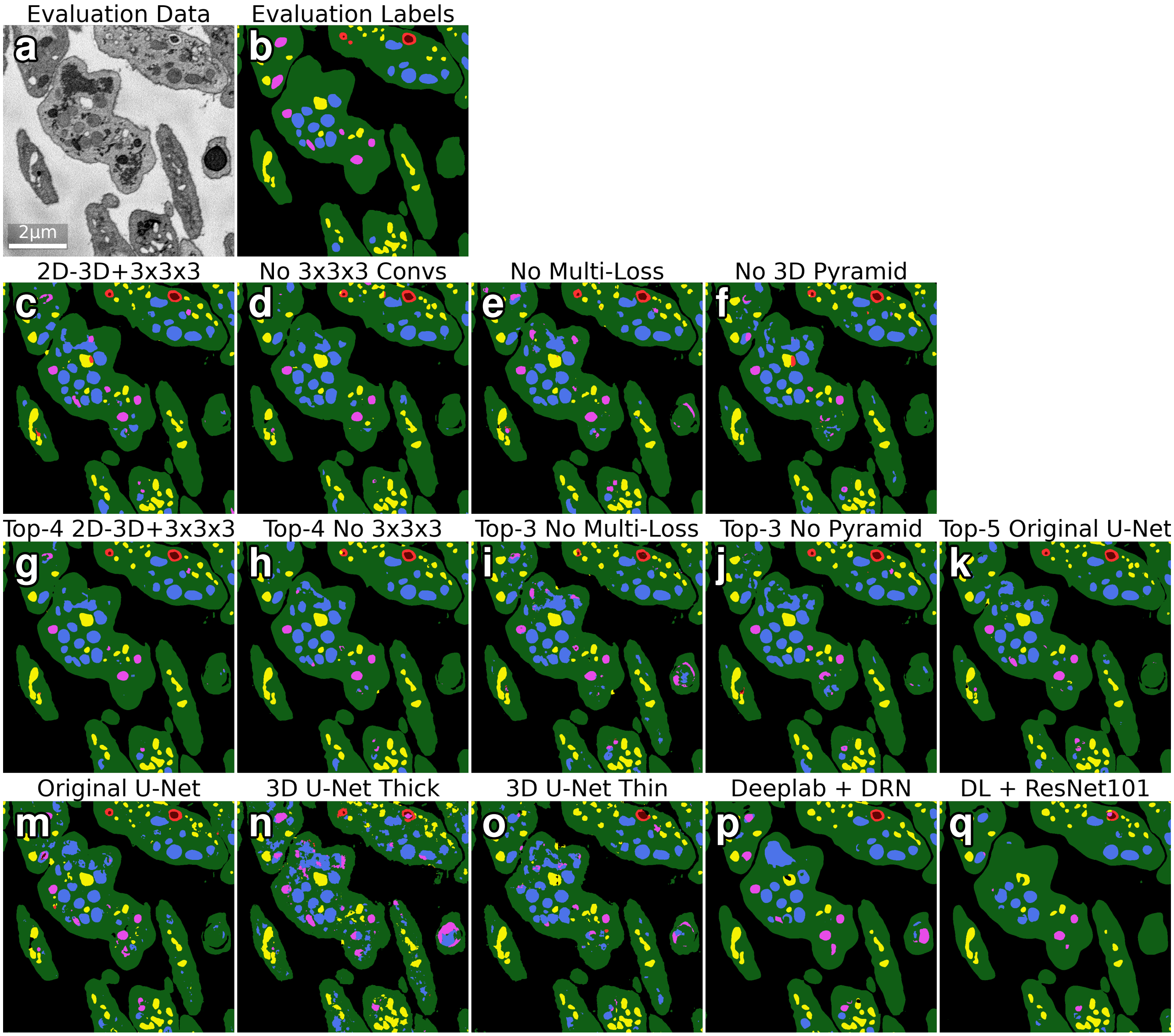

Figure S2. **2D comparison of all algorithm results**. This figure compares the results of all 14 segmentation algorithms tested in this paper with ground-truth labels for the $z = 4$ slice of the evaluation dataset. (**a**-**b**) Orthoslice of the evaluation image dataset and segmentation. (**c**-**f**) Segmentations from our new 2D-3D+3x3x3 network and its three ablations. (**g**-**k**) Segmentations from the five ensemble algorithms tested in this work. (**m**-**q**) Segmentations from the five baseline networks tested in this work.

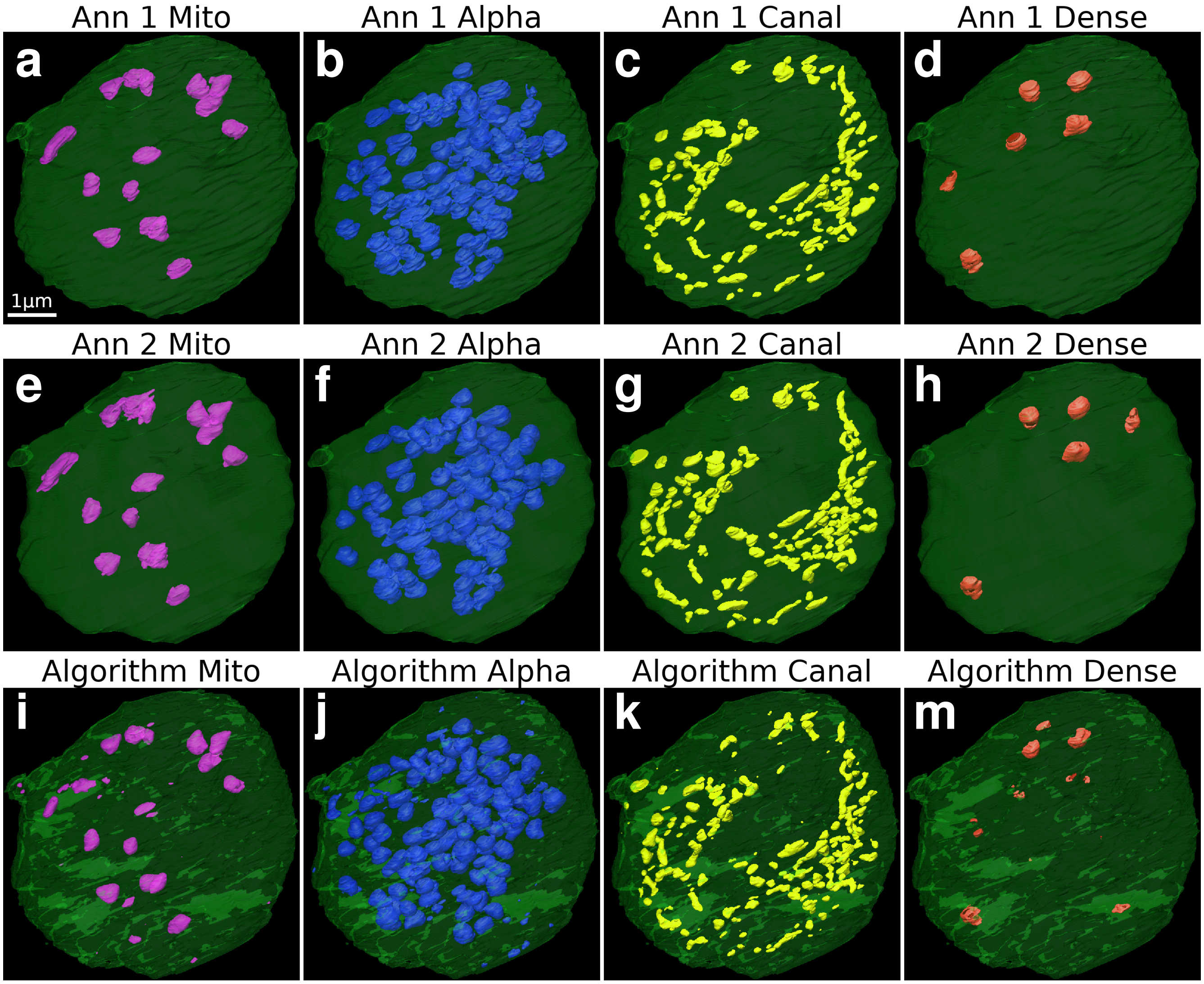

Figure S3. Annotator comparison segmentation renderings. This figure supplements row 4 of Figure 3 by showing renderings of individual organelle types - Mito for mitochondria, Alpha for alpha granules, Canal for canalicular channels, Dense for dense granules - from the Annotator 1, Annotator 2, and best Algorithm (Top-4 2D-3D+3x3x3) segmentations. (**a**-**d**) Annotator 1 (Ann 1) organelle segmentations. (**e**-**h**) Annotator 2 (Ann 2) organelle segmentations. (**i**-**m**) Algorithm organelle segmentations.

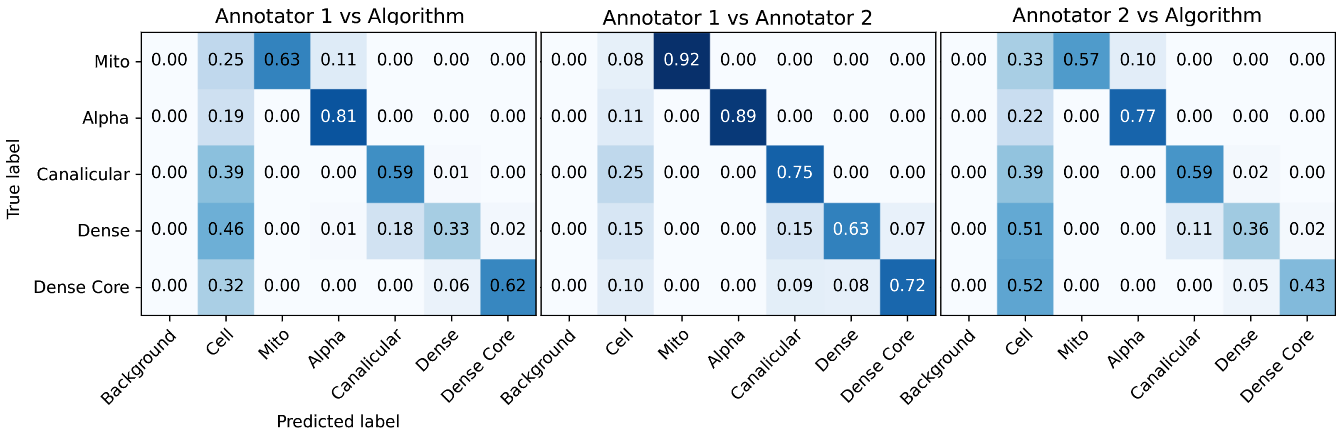

Figure S4. Annotator comparison confusion matrices. Confusion matrices comparing organelle labelings pairwise between the two annotators and our best algorithm. These give a more detailed performance breakdown of the MIoU$^{(org)}$ scores obtained for each comparison: 0.497 for Annotator 1 vs Algorithm, 0.571 for Annotator 1 vs Annotator 2, and 0.483 for Annotator 2 vs Algorithm.

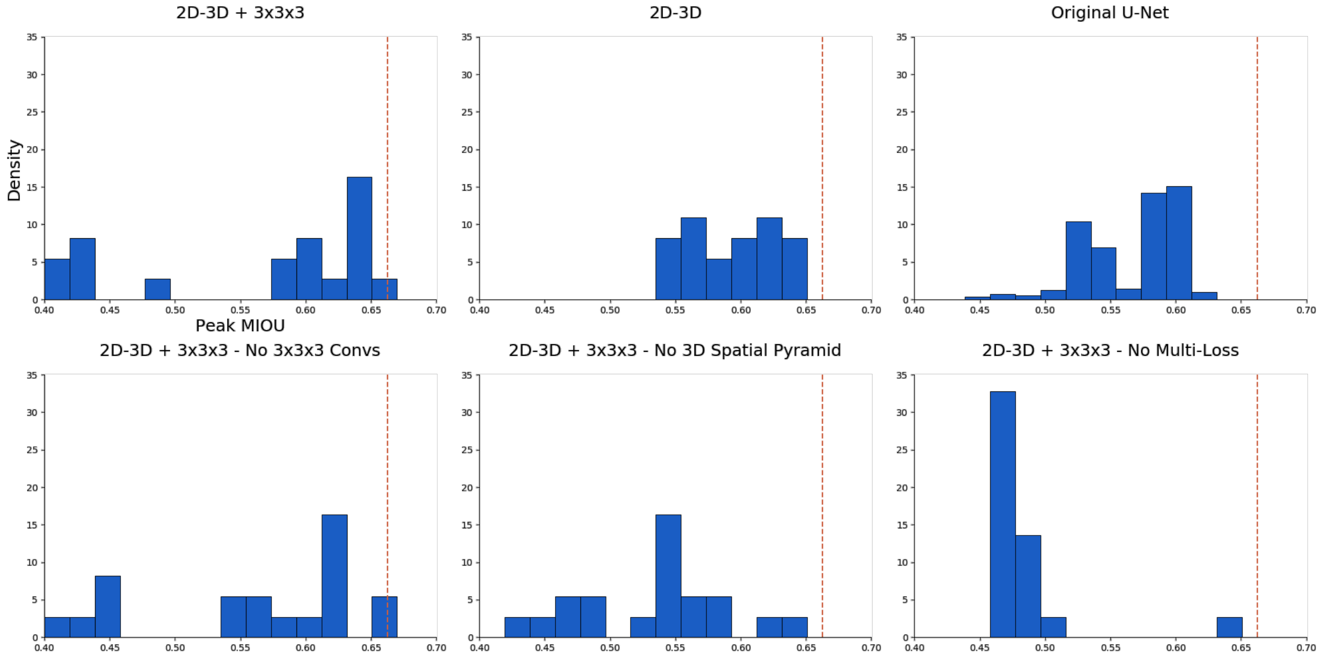

Figure S5. Making better architecture design decisions. This figure shows normalized histograms of peak MIoU$^{(all)}$ on the evaluation dataset for each of the architectures examined in this paper. In order to determine whether one architecture choice is superior to another, the outputs of different trained networks are compared with each other. However, sources of randomness in the training process (initialization of trainable weights from a Xavier uniform distribution, and the random presentation order of training data elements) induce a distribution of final performance metric scores. These scores are random variables, and a single sample per architecture may be insufficient to determine which is better. By empirically approximating the distribution for each architecture, better inferences may be made about architecture design choices. For this figure, multiple instances of the same architecture (26 for 2D-3D nets, 500 for the U-Net) were trained under identical conditions, varying only random number generation seeds. The resulting distributions support the conclusions that 2D-3D networks outperform the 2D U-Net and that multi-loss training is necessary for 2D-3D architectures.

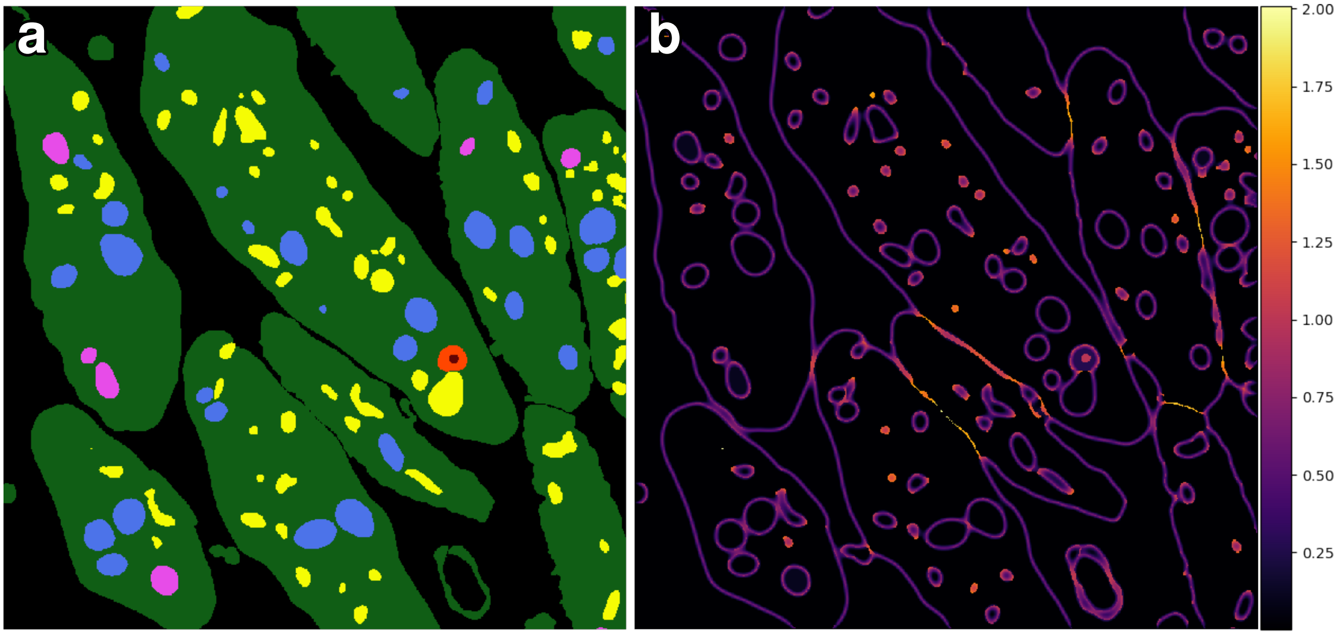

Figure S6. Error weighting array visualization. The error weighting $\mathcal{W}$ array is the sum of three terms $w + \mathcal{W}_{cb} + \mathcal{W}_{ep}$, where w is a weight floor, $\mathcal{W}_{cb}$ is a class frequency balancing array, and $\mathcal{W}_{ep}$ is an edge preserving array. $\mathcal{W}_{cb}$ and $\mathcal{W}_{ep}$ are computed from ground truth labels. (**a**) Example orthoslice of training dataset ground truth labels. (**b**) Corresponding orthoslice of the error weighting array.

	Eval MIoU$^{(all)}$	Eval MIoU$^{(org)}$	Test MIoU$^{(org)}$
Top-4 2D-3D+3x3x3	0.686	0.595	**0.446**
Top-5 No 3x3x3 Convs	**0.690**	**0.601**	0.419
Top-3 No Multi-Loss	0.633	0.524	0.338
Top-3 No 3D Pyramid	0.681	0.590	0.421
Top-5 Original U-Net	0.663	0.562	0.371

(a) Ensembles of Networks

2D-3D+3x3x3 (10.3M)	0.665	0.568	0.417
No 3x3x3 Convs (9.9M)	0.667	0.571	0.358
No Multi-Loss (10.3M)	0.652	0.550	0.355
No 3D Pyramid (7.9M)	0.646	0.542	0.376

(b) Single 2D-3D+3x3x3 Network and Ablations

Original U-Net (31.0M)	0.626	0.515	0.334
3D U-Net Thick (2.1M)	0.496	0.348	0.314
3D U-Net Thin (2.0M)	0.613	0.502	0.280
Deeplab + DRN (40.7M)	0.632	0.522	0.130
Deeplab + ResNet101 (59.3M)	0.585	0.456	0.124

(c) Baseline Networks

Table S1. Comprehensive network performance statistics. Segmentation algorithm results summary showing mean intersection-over-union (MIoU) across all classes (MIoU$^{(all)}$) on evaluation data and MIoU across organelle classes (MIoU$^{(org)}$) on evaluation and test data. The Subject 2 dataset from which the test data is taken contains only a small number of labeled cells among unlabeled ones; we use MIoU$^{(org)}$ to measure test performance since restricting the MIoU stat to labeled regions invalidates `background` and `cell` class statistics. (**a**) Results for the best ensemble from each architecture tested. A top-*k* ensemble averages the predictions of the best *k* trained networks as judged by MIoU$^{(all)}$ on the evaluation dataset. (**b**) Results for the best single network from each architecture class. Trainable parameter counts are in parentheses. (**c**) Results from baseline comparison networks. Trainable parameter counts are in parentheses
